# A Case Report: Hypoglycaemic Unawareness Associated With Insulinoma

**DOI:** 10.7759/cureus.64994

**Published:** 2024-07-20

**Authors:** Alamin Alkundi, Rabiu Momoh

**Affiliations:** 1 Diabetes and Endocrinology, East Kent Hospitals University Foundation Trust, Kent, GBR; 2 Critical Care, William Harvey Hospital, Ashford Kent, GBR

**Keywords:** pancreatic enucleation, pro-insulin, insulin, continuous glucose monitoring, c-peptide, pancreatic disorder, hyperinsulinism, hypoglycaemia, hypoglycaemic unawareness, insulinoma

## Abstract

Hypoglycaemic unawareness (HU) is more frequently described in relation to diabetics in the literature. We have noted that there is also an increasing reporting of HU in insulinoma cases. We report a hospital presentation for an incidental finding of hypoglycaemic unawareness in a gentleman in his fifties who was eventually diagnosed with insulinoma following biochemical studies, radiologic evaluation and histologic evaluation of an excised lesion between the pancreas and the spleen. We have reviewed existing literature evidence regarding the possible aetiologies and management options for this occurrence. More research studies to identify the epidemiology of this association and the determination of a protocol for increased detection of patients with insulinoma who display HU will need to be done.

## Introduction

Hypoglycaemic unawareness, a condition of being oblivious to warning symptoms of low blood sugar readings, could potentially be harmful, tipping patients into loss of consciousness, seizures, cardiac arrhythmias, and possibly fatality. Humans experience neurologic and autonomic symptoms/signs in response to low blood sugar readings as a protective mechanism. The blunting of the aforementioned responses or their complete absence has been majorly described in drug-treated diabetics (more common in Type I diabetics than in Type II diabetics). There is an increasing description in the literature of hypoglycaemic unawareness in patients who go on to be diagnosed with insulinoma [1].

Insulinoma is a neuroendocrine tumour that may be found in the pancreas or extra-pancreatic and causes hypoglycaemia. Other important differential diagnoses to consider in the assessment and management of hyper-insulinemic hypoglycaemia include factitious hypoglycaemia, postprandial dumping syndromes, hypopituitarism, insulin receptor gene mutations, and chronic adrenal insufficiency [2]. The prevalence rate of insulinoma is one to four persons per million of the general population. Insulinoma is diagnosed clinically by the presence of Whipple's triad (hypoglycaemic symptoms, demonstration of low blood sugar (<50 mg/dl) and reversal of the hypoglycaemic symptoms by intravenous glucose correction). A three-day fasting test and a later demonstration of endogenous hyper-insulinemic hypoglycaemia, along with raised C-peptide levels, are also used in the assessment of insulinoma [3].

With this case report, we aim to highlight the danger of hypoglycaemic unawareness (and all its attendant severe consequences) pre-dating a diagnosis and an eventual treatment for insulinoma in patients. We hope to highlight the existing knowledge gap in the early identification of this subset of patients with insulinoma who experience hypoglycaemic unawareness, as most referenced cases reviewed along with our index case were incidentally uncovered.

## Case presentation

A male patient in his fifties, a farmer by occupation, had a wellness check and was found with an incidental low blood glucose level for which he was asymptomatic. Repeat capillary blood glucose (CBG) at his general practitioner's office was 2.6 mmol/l; still, the patient had no hypoglycaemic warning symptoms. Initial treatment was offered for the hypoglycaemia, and the patient was then referred to the hospital for further investigations.

The patient reported being usually well in health (with nil significant past medical history) and was usually physically active (working on a farm). On assessment in the hospital, the patient was noted to have no hypoglycaemic warning symptoms, even with extremely low blood sugar readings. He had no recurrent dizziness or diaphoresis. He had no polyphagia/polyuria or polydipsia. He did not feel more hungry than usual. He noted no recent weight changes. He had no family history of diabetes, thyroid disease, auto-immune diseases, or cancer. The patient was not on any regular medication or over-the-counter medications. He was a non-smoker and did not consume alcohol. He had no known drug allergy. The patient did not consume any unusual foods, such as Ackee, which could precipitate hypoglycaemia. He had undergone an inguinal herniorrhaphy surgery 20 years prior. His vital signs were within normal limits, and a general and abdominal examination conducted on him was unremarkable. HbA1C (glycated haemoglobin) checked while in the hospital was 21.5 mmol/mol (ref: < 42 mmol/mol), excluding diabetes mellitus. The patient’s pre-meal blood glucose readings were around 3-3.5 mmol/L, then after meals, they did not go above 4.5 mmol/L. A short Synacthen test showed adequate response with a basal cortisol of 347 nmol/l and a post-Synacthen cortisol value of 712 nmol/l, excluding Addison's disease as the cause of the patient's recurrent hypoglycaemia.

**Figure 1:**
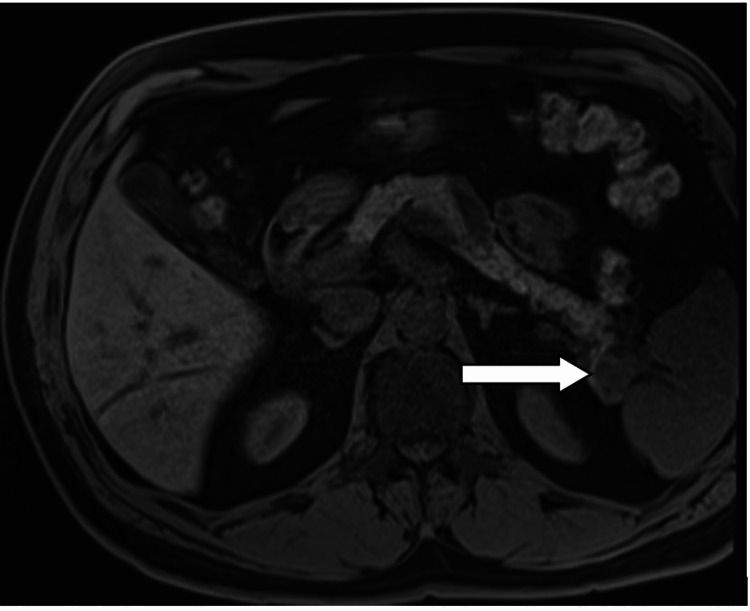
MRI abdomen transverse section revealing a 2.3 x 2.0 cm-sized lesion adjacent to the splenic hilum and tail of the pancreas

An MRI abdomen was done in a bid to identify the culprit insulinoma lesion (Figure 1). A well-defined, 2.3 x 2.0 cm sized, enhancing lesion adjacent to the splenic hilum and tail of the pancreas that enhanced more than the spleen on postcontrast images was seen. Multiple T2 hyperintense lesions were noted within the spleen, the largest in the lower pole of the spleen, measuring 2.0 x 1.7 cm. Some of the splenic lesions showed thin peripheral enhancement on postcontrast images. The liver, gallbladder and adrenal glands were unremarkable. Small cortical cysts were noted in both kidneys.

An MRI head was done in a bid to exclude any pituitary lesion, and this yielded no acute finding. The normal size of the sella was preserved. The pituitary glandular parenchyma appeared unremarkable and demonstrated no evidence of any abnormal/differential enhancement in the post-contrast images to suggest a microadenoma. The pituitary stalk was central and not displaced. Normal appearances of the sphenoidal sinus, suprasellar cistern, optic chiasma, hypothalamus, and cavernous sinuses were seen, and cavernous segments of the bilateral internal carotid artery were preserved.

The patient was educated about a possible diagnosis of insulinoma based on his clinical features, laboratory study and radiologic findings. He was provided with Freestyle Libre (Abbott Laboratories, Green Oaks, Illinois, US) for self-glucose monitoring and was recommended for a repeat prescription of this by his general practitioner upon discharge. He was also supplied with a rescue medication pack containing intramuscular glucagon and per-oral glucose preparation for use as required upon discharge from the hospital. The patient was given safe-netting advice, and red flag signs were explained. A hypoglycaemia leaflet was provided to him. It was recommended that he get input from community dietetics as part of follow-up care.

The patient later underwent an outpatient mixed-meal test. An intravenous cannula was inserted, and baseline glucose levels were taken. He was then given Fortisips, an energy drink to ingest over five minutes. Blood glucose levels were then taken every 30 minutes for 5 hours (Table 1). The C-peptide level was taken at a point when the capillary blood glucose level was 2.0 mmol/l, and the result was 4333 pmol/l (elevated) and blood insulin level 562 pmol/l (18-173 pmol/l). The plasma sulfonylurea screen in this patient was negative to exclude the factitious cause of hyper-insulinemic hypoglycaemia in this patient. Immunoglobulin G (IgG) insulin antibody was 13.4 mg/L (positive). The results of other neuroendocrine tumour markers screened for are provided in Table 2. He commenced on daily oral diazoxide 300 mg OD after these study results and was referred to a tertiary endocrinology service.

**Table 1:** Results from the mixed meal tolerance test conducted on the patient Serum C-peptide and insulin level results are also shown.

	0	30 mins	60 mins	90 mins	120 mins	150 mins	180 mins	210 mins			240 mins	270 mins	300mins
Capillary blood glucose (Ref 2-5.4 mmol/l)	2.7	4.6	5.2	5.1	5.8	6.6	5.8	2.0			1.9	2.2	2.5
C-peptide (370-1470 pmol/l)									4333				
Insulin (Ref 18-173 pmol/l)										562			

**Table 2:** Neuroendocrine tumour markers screen results

Tests	Reference ranges	Results
Urine 5-HIAA (random)	< 4 micromol/mmol	1.7
Urine creatinine	3.5 - 24.6 mmol/l	20
Vasoactive intestinal peptide	< 30 pmol/l	9
Pancreatic polypeptide	< 300 pmol/l	149
Glucagon	< 50 pmol/l	24
Somatostatin	< 150 pmol/l	22
Gastrin	< 40 pmol/l	7
Chromogranin A	< 60 pmol/l	19
Chromogranin B	< 150 pmol/l	57

A multidisciplinary review of this patient at the tertiary centre agreed that the patient’s case was possibly a case of malignant insulinoma based on the biochemical studies and possible radiologic findings. He underwent a DOTATATE PET/CT study at the tertiary centre, revealing a moderate to low-grade somatostatin expression within the soft tissue nodule adjacent to the pancreatic tail and splenic hilum. He was recommended to continue the use of per-oral diazoxide 100 mg B.D. pending when endoscopic ultrasound and possible surgery would be done. An endoscopic ultrasound was eventually done but a biopsy of the identified lesion was not done due to the presence of surrounding vascular structures and the risk of major bleeding. The patient then underwent laparoscopic distal pancreatectomy and splenectomy under general anaesthesia.

Pathological study of the excisional biopsy revealed a tumoral nodule at the very tail of the pancreas that was well-demarcated, measuring 2.6 cm x 1.8 cm x 2.1 cm, and was a well-differentiated neuroendocrine tumour (insulinoma), G2 (Ki67 5.1%), 26 mm, completely resected. There was no infiltration of the adjacent adipose tissue, splenic parenchyma, splenic artery and splenic vein. TNM (tumour node metastases) staging (Union for International Cancer Control (UICC) 8th edition): pT2, pN0 (0/16), LV0, R0. On microscopy, the tumour cells were cuboidal to columnar, in a predominantly trabecular pattern with rich vasculature. The tumour cells show eosinophilic granular cytoplasm, and round/oval mildly pleomorphic nuclei with granular chromatin. Tumour cell density was variable with areas of the lesion showing attenuation of the trabecules by eosinophilic amorphous stroma. Tumour necrosis was not a feature. On immunochemistry, the tumour cells expressed ISL1 protein and insulin. There was no tumoral involvement with the spleen.

Continuous blood glucose monitoring was done over a two-week period and revealed an improvement in his blood glucose readings with fasting and post-prandial readings ranging between 5 and 10 mmol/l. The patient was recommended for a review in the clinic three to six months post-surgery to exclude the recurrence of the tumour with an MRI abdomen and blood glucose assessment.

## Discussion

We have presented the case of a 55-year-old male with hypoglycaemic unawareness (incidentally discovered), who went on to be diagnosed with insulinoma and underwent a laparoscopic resection of a lesion (insulinoma) between the pancreas and splenic hilum. There was an improvement in the patient's blood sugar readings to normal values post-surgery.

Fasting hypoglycaemia occurs in 80% of patients with insulinoma [4]. The epidemiology of hypoglycaemic unawareness in insulinoma is yet to be well-described. Nakajima R et al. (2023) reported a likely period of five years with possible hypoglycaemic unawareness in a 66-year-old male who went on to be diagnosed with insulinoma after he presented to the hospital with symptoms suggesting eventual awareness of hypoglycaemia. Their patient was identified five years prior with low blood glucose readings with no neuroglycopenic symptoms or signs while blood studies (done four hours post-meal) for assessment of his hypertension were being done [5]. Lo CH et al., in a 2016 case report, described the finding of low blood sugar readings, a significantly low insulin level, and elevated C-peptide in a 50-year-old female patient who went on to have a histologically diagnosed insulinoma from a laparoscopic distal pancreatectomy. They suggested that contradictory levels of insulin and C-peptide should not exclude making a diagnosis of insulinoma. They also proposed that a 24-hour fast test has the same clinical significance as the 72-hour fast test in the assessment of insulinoma cases. They also described the presence of hypoglycaemic unawareness after overnight fasts in their patient [6]. In our index case report, our patient had hyper-insulinemic hypoglycaemia, had raised C-peptide levels, had a positive IgG insulin antibody test, as well as experienced hypoglycaemic unawareness.

Computerised tomography or magnetic resonance imaging may be used for non-invasive localization of insulinoma while endoscopic ultrasound (EUS) of the pancreas and intra-arterial injection-venous blood sampling can be used for invasive localisation of insulinomas where surgery is being planned. The definitive diagnosis of insulinoma is via histology. Surgical intervention (resection of insulinomas) achieves a high cure rate in a lot of benign insulinoma cases. Other treatment methods suggested in benign insulinoma cases include injection of octreotide, EUS-guided ablation with alcohol, radiofrequency ablation (RFA), or embolization of a pancreatic insulinoma. Aggressive resection may be offered in malignant insulinoma cases that show local or lymph node spread. Hepatectomy and possibly liver transplantation can be considered where there is hepatic involvement. RFA, embolization, intra-arterial chemotherapy (in hepatic metastasis) or continuous glucose monitoring plus medical treatment could be offered in unresectable malignant insulinoma cases [3].

The index patient being reported was treated with diazoxide while attempts were being made to localize the culprit lesion for consideration of surgery. Pathological studies done on excised tissues confirmed insulinoma as a diagnosis histologically.

A wider description of hypoglycaemic unawareness in the literature is in diabetics (in Type I diabetics more than Type II diabetics). Advanced ages, patients with a long diabetic history and patients on intensive diabetic treatment are also thought to be at higher risks of developing hypoglycaemic unawareness. Several mechanisms have been suggested as aetiologies for hypoglycaemic unawareness, and these include protracted or recurrent exposures to low blood sugar levels and failure of counter-regulatory hormones (such as glucagon, catecholamines, growth hormone and cortisol). There is, however, an increasing literature description of HU in insulinoma patients [5]. Christesen HB et al. (2008), in another vein, described a unifying finding of hypoglycaemic unawareness among four relatives with a rare condition called non-insulinoma persistent hyperinsulinaemic hypoglycaemia (NI-PHH) that is caused by an activating glucokinase mutation [7].

In a 1993 publication by Mitrakou A et al., they demonstrated that there was generally a decreased counter-regulatory hormone level (plasma catecholamines, glucagon, growth hormone, and cortisol) in insulinoma patients when they conducted stepped hypoglycaemic-clamp studies in 6 insulinoma patients when compared to 14 normal subjects (matched for age, weight and sex). They noted that there was a reversibility of the counter-regulatory hormone levels approximately six months after curative surgeries were done. They also noted that patients with insulinomas who were untreated had reduced autonomic and neuroglycopenic symptoms, as well as reduced deterioration of cognition during hypoglycaemia [1].

In a 2021 publication by Nosáková L et al., detailing the outcome of a retrospective analysis of 22 cohort cases of insulinoma (confirmed with 72 hrs fasting tests), they found seven cases (31.8%) among their study population that demonstrated hypoglycaemic unawareness. They found that immune-reactive insulin (IRI) (2.35 ± 1.25 vs. 5.88 ± 3.92 ng/ml, p=0.01) and C-peptide (9.14 ± 7.36 vs. 50 ± 42.8 µU/ml, p=0.01) were significantly lower than the rest of the insulinoma patients who did not have hypoglycaemic unawareness. During the fasting test, nadir blood glucose level did not show any significant statistical difference (9.4 ± 8.2 vs. 12.2 ± 8.2 months, p=0.28) in the two groups of patients. The mean age of the patients they studied was 51 ± 16.7 years [8].

Matsumoto K et al., in a 2023 case report, described the post-partum diagnosis of insulinoma in a 40-year-old female patient who had an episode of hypoglycaemic coma in the third month of pregnancy (blood glucose level of 36mg/dl) that was then attributed to hyperemesis gravidarum. There were no further hypoglycaemic episodes noted in their patient for the remainder of her pregnancy. A 75-g oral glucose tolerance test was done during week 33 of pregnancy, and no abnormalities were observed. The patient was delivered a 2.595 kg baby at week 38 of pregnancy via Caesarean section. The patient was found in a hypoglycaemic coma on day 3 (with a blood sugar level of 16 mg/dl). No pancreatic tumour was found in contrast-enhanced CT, gadolinium-enhanced magnetic-resonance imaging (Gd-MRI) or somatostatin receptor scintigraphy studies done. A positive selective arterial calcium stimulation test was then done, and a further assessment with an endoscopic ultrasound study revealed a 0.7 cm low-echoic pancreatic body mass. The patient then had an open spleen-preserving distal pancreatectomy surgery done, aided with ultrasound guidance. Matsumoto K et al. mentioned that possibly pregnancy-associated enhancement of insulin resistance prevented further hypoglycaemic episodes in their patient after the initial hypoglycaemic episode at three months of gestation. The repeat hypoglycaemic coma in the post-partum period would possibly have timed the normalization of insulin resistance after pregnancy, hence the unmasking of the effect of the insulinoma [9]. The potential role of hypoglycaemic unawareness as a cause of the repeated hypoglycaemic coma in the insulinoma case described above remains a possibility.

Ferreira AI et al. (2020) described an eventual assessment of hypoglycaemic unawareness due to possible insulinoma in a 78-year-old female who was initially being managed in the hospital for infected bronchiectasis and was in type 1 respiratory failure. Previous hospital admissions had noted repeated hypoglycaemic recordings but were excused to be due to the acute exacerbation of the patient's chronic health condition. She underwent assessments that confirmed the presence of hyperinsulinism and raised C-peptide. A wide area of localization of insulin hyperproduction was found in the body and tail of the patient's pancreas, and a conservative treatment approach with diazoxide was decided on after factoring in the patient's co-morbidities and the high surgical risk involved [10].

Aida A et al. (2022) described in a case report the use of a continuous blood glucose monitoring device along with the administration of diazoxide as an interim treatment option to manage patients with hypoglycaemic unawareness caused by insulinoma while awaiting definitive surgical treatment option [11]. The above-described measure was undertaken in the management of our index case that is being reported. Nakajima R (2023) alluded to the usefulness of continuous blood glucose monitoring devices in assessing the outcome of surgical intervention for cases of insulinoma, as well as being useful for the pre-operative evaluation of hypoglycaemia in these cases [12].

Complete avoidance of hypoglycaemia is the major step to prevent hypoglycaemic unawareness in diabetics, and this is often challenging to achieve. Blood glucose monitoring, setting individualized targets and education are some of the means available to achieve the above-stated goals. The association of hypoglycaemic unawareness with insulinoma cases is on the increase. Minimizing the impact of this on insulinoma patients should be the goal via a heightened awareness of this association by managing clinicians, facilitating early confirmation of an insulinoma diagnosis and the institution of definitive treatment.

## Conclusions

More research into the aetiology and epidemiology of hypoglycaemic unawareness in insulinoma cases is suggested. The potential delay in the time to diagnosis of insulinoma in those with hypoglycaemic unawareness increases morbidity and mortality risks for these patients. Our case report adds to the evidence regarding the role of continuous blood glucose monitoring and medical treatment with diazoxide in the interim management of patients with clinically suspected insulinoma awaiting tumour localization investigations and then surgery. We have also alluded to the role of the use of continuous glucose monitoring to evaluate the outcome of surgery in these patients.
